# Bifidobacteria grown on human milk oligosaccharides downregulate the expression of inflammation-related genes in Caco-2 cells

**DOI:** 10.1186/s12866-015-0508-3

**Published:** 2015-08-25

**Authors:** Saumya Wickramasinghe, Alline R. Pacheco, Danielle G. Lemay, David A. Mills

**Affiliations:** Department of Basic Veterinary Sciences, University of Peradeniya, Peradeniya, 20400 Sri Lanka; Foods for Health Institute University of California, Davis, Davis, CA 95616 USA; Department of Food Science and Technology, University of California, Davis, Davis, CA 95616 USA; Genome Center, University of California, Davis, Davis, CA 95616 USA; Department of Viticulture and Enology, University of California, Davis, Davis, CA 95616 USA

## Abstract

**Background:**

Breastfed human infants are predominantly colonized by bifidobacteria that thrive on human milk oligosaccharides (HMO). Two predominant species of bifidobacteria in infant feces are *Bifidobacterium breve (B. breve)* and *Bifidobacterium longum* subsp*. infantis* (*B. infantis*), both of which include avid HMO-consumer strains. Our laboratory has previously shown that *B. infantis*, when grown on HMO, increases adhesion to intestinal cells and increases the expression of the anti-inflammatory cytokine interleukin-10. The purpose of the current study was to investigate the effects of carbon source—glucose, lactose, or HMO—on the ability of *B. breve* and *B. infantis* to adhere to and affect the transcription of intestinal epithelial cells on a genome-wide basis.

**Results:**

HMO-grown *B. infantis* had higher percent binding to Caco-2 cell monolayers compared to *B. infantis* grown on glucose or lactose. *B. breve* had low adhesive ability regardless of carbon source. Despite differential binding ability, both HMO-grown strains significantly differentially affected the Caco-2 transcriptome compared to their glucose or lactose grown controls. HMO-grown *B. breve* and *B. infantis* both downregulated genes in Caco-2 cells associated with chemokine activity.

**Conclusion:**

The choice of carbon source affects the interaction of bifidobacteria with intestinal epithelial cells. HMO-grown bifidobacteria reduce markers of inflammation, compared to glucose or lactose-grown bifidobacteria. In the future, the design of preventative or therapeutic probiotic supplements may need to include appropriately chosen prebiotics.

**Electronic supplementary material:**

The online version of this article (doi:10.1186/s12866-015-0508-3) contains supplementary material, which is available to authorized users.

## Background

Milk is a unique biological fluid consumed by mammalian infants. It contains many macro- and micro-nutrients that are essential for the growth and development of the newborn [1, 2]. In addition, a diverse cocktail of bioactive factors, such as oligosaccharides, antibodies and nucleotides in milk, play immune, prebiotic and protective functions in the infant gut [2–4]. Oligosaccharides are the third most abundant component in human milk and they are present as lactose-derived free forms and protein and lipid bound glycoconjugates [5]. Milk oligosaccharides can withstand the pH of the stomach and virtually all of them resist enzymatic digestion in the gastro-intestinal tract [6]. Recent studies on human milk oligosaccharides (HMO) and glycoconjugates demonstrate both local and systemic beneficial effects to the suckling neonate [7–9]. Milk oligosaccharides provide protection against enteric pathogen infections by antibacterial activity, competing with pathogen binding sites and enhancing the binding of IgA with pathogens [10]. Another protective function of milk oligosaccharides is that the intact oligosaccharides serve as a prebiotic substrate enabling enrichment of *Bifidobacterium* species in the infant gut thereby consuming available nutrients and lowering the gut pH [11].

*Bifidobacterium* species were first observed over 100 years ago in feces of breastfed infants and later research suggested breast milk contains special molecules defined as “bifidus factors” that stimulate the growth of bifidobacteria [12, 13]. Culture-based studies over the years and high-throughput metagenomic studies have demonstrated that *Bifidobacterium* is a commonly enriched member of the intestinal microbiota of breastfed infants [14, 15]. Research in the last decade has provided a mechanistic basis for that enrichment whereby HMOs and glycoconjugates serve as prebiotics selectively promoting bifidobacteria [16]. Indeed, genomic analysis of a prototypical infant borne bifidobacteria, *Bifidobacterium longum* subsp. *infantis* (*B. infantis*) which grows well on HMO, revealed a single cluster of genes encoding milk oligosaccharide metabolism suggesting co-evolution of this strain with human milk [17, 18]. Analysis of other infant-borne strains of *B. bifidum*, *B. longum* subsp. *longum* (*B. longum*) and *B. breve* shown to grow on HMO [19, 20] also possessed specific milk glycan transporters and glycosyl hydrolases linked to milk glycan consumption [16, 19].

The ability of bifidobacteria to bind and interact with the intestinal epithelium plays an important role in gut colonization and modulation of host immune system [21, 22]. Previous research has proven that different species of bifidobacteria exhibit different capacities to adhere to the intestinal epithelium and to stimulate the gastrointestinal immunity [23–25]. Our research group recently showed that the adhesion rate of bifidobacteria to the intestinal epithelial cells (IECs) changes according to the carbon source supplied in their growth medium [26]. When comparing *B. infantis* ATCC 15697 grown in HMO and lactose, the HMO grown *B. infantis* had a significantly higher rate of adhesion to both Caco-2 and HT-29 cells [26]. This work was subsequently confirmed by Kavanaugh et al. showing that growth of *B. infantis* ATCC 15697 on 6’sialyllactose (an HMO component structure) also resulted in increased adherence to cultured IECs [25]. Previous research also showed the ability of bifidobacteria to induce the anti-inflammatory capacity of IECs. For example, colon organ cultures exposed to *B. infantis* showed reduced production of pro-inflammatory cytokine IL-17 [27]. In another study, interferon gamma was reduced in the Peyer’s patches of mice fed *B. longum* [28].

There are few documented studies on changes in the interaction between bifidobacteria and IECs as a function of the carbon source of the bacterial growth medium. Microarray studies in our laboratory have previously shown that incubation of HMO with *B. infantis* altered gene expresssion in Caco-2 cells [29]. Chichlowski et al. observed that HMO-fed *B. infantis* tightens cell-cell junctions, increases the level of cytokine IL-10 while decreasing the level of pro-inflammatory TNFα in Caco-2 cells [26].

In the current study, we sought to understand the effects of HMO-fed bifidobacteria on the gene expression of intestinal cells on a genome-wide basis. We investigated the effects of two bifidobacterial strains fed different carbon sources—HMO, glucose (GLU), or lactose (LAC)—on the gene expression of Caco-2 cells using RNA sequencing (RNA-Seq). Due to their dominance in breastfed infant feces [30] and efficient consumption of HMOs, *B. infantis* and *B. breve* were selected for the study. The two strains selected were *B. infantis* ATCC 15697, which is the current model to study genetic adaptations to growth on human milk [31] and *B. breve* SC95, a strain that also grows vigorously on HMOs [19]. Our group has also shown that HMO consumption by *B. infantis* triggers expression of surface binding proteins that interact with intestinal cell surface structures [32]. We hypothesized that HMO-grown bifidobacteria, relative to those grown on LAC or GLU will have enhanced adhesion and will alter gene expression in Caco-2 cells consistent with a protective modulatory mechanism in the host intestine.

## Results

### Adhesion of bifidobacteria grown in different carbon sources to Caco-2 cells

**Fig. 1:**
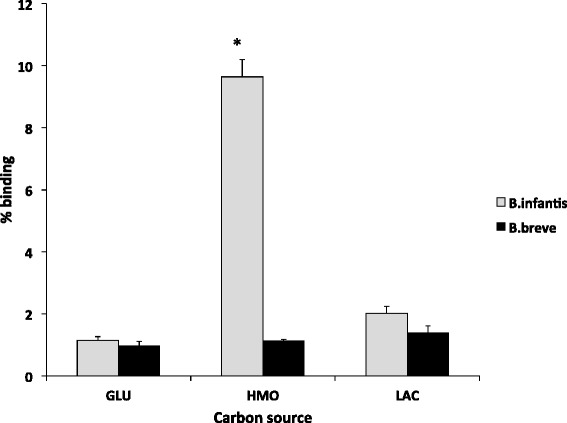
Binding of *B. breve* SC95 and *B. infantis* ATCC 15697 to Caco-2 cell monolayers, expressed as percentage of initial bacterial input. Statistical analysis was performed by ANOVA, and binding percentages were compared to LAC. * *P* < 0.01

Several studies have demonstrated the ability of *B. infantis* strains to grow on HMO as the sole carbon source [33] and a recent study in our laboratory identified three strains of *B. breve* (SC95, SC154, and ATCC 1570) that can also efficiently consume HMO [19]. Based on the results of this study and their exclusive presence in infant feces, *B. breve* SC95 was selected for the adhesion assay. Different adhesion percentages were observed between the two bifidobacterial species. The levels of adhesion in *B. infantis* ATCC 15697 ranged from 1.1 to 9.6 % and in *B. breve* SC95 it ranged from 0.9 to 1.3 % (Fig. 1). Binding percentages obtained for *B. infantis* ATCC 15697 were very similar to that reported previously [25, 26]. However, the current study included the GLU-grown *B. infantis* ATCC 15697, which showed the lowest percentage of adhesion. Compared to *B. infantis* ATCC 15697, *B. breve* SC95 presented lower binding efficiency to Caco-2 cells. The highest binding percentage (1.3 %) of *B. breve* SC95 was observed for LAC-grown bacteria and there were numerical trends among treatments and the adhesion efficiency, however no statistical significant differences were observed among them. Studies of different *B. breve* strains grown in MRS media have shown very low adhesion percentages to Caco-2 cells [34]. In the current study, we have observed lower binding of *B. breve* SC95, regardless of the carbon source.

### Gene expression analysis of Caco-2 cells in response to bifidobacteria grown on different substrates

In order to examine if bifidobacteria grown in different carbon sources elicit a change in the intestinal cellular responses, we examined the transcriptome of the Caco-2 cells using RNA-Seq. This resulted in 20–32 million reads per sample and only uniquely mapped reads were considered in the analysis. As described by Bentley et al. [35] and Ramskold et al. [36] a threshold RPKM (reads per kilo base per million mapped reads) value of 0.1 RPKM was established to balance the number of false positives and false negatives and to define potential meaningful gene expression. There were 15,613, 15,574, and 15,520 genes expressed in Caco-2 cells exposed to *B. breve* SC95 grown in GLU, HMO and LAC respectively. Caco-2 cells exposed to *B. infantis* grown in GLU, HMO and LAC had 15,561, 15,468 and 15,516 expressed genes respectively. Summaries of gene expression intensities for all transcripts in all samples are provided in the NCBI GEO repository, accessions GSE63950 and GSE64017.

### Effect of carbon source consumption by bifidobacteria on Caco-2 gene expression

To determine the specific effect of carbon source on Caco-2 gene expression, differential expression between carbon sources were analyzed individually in each bifidobacteria strain experiment. As expected, bifidobacteria grown in different carbon sources elicited significant changes (*p*-value ≤0.05, FDR q ≤ 0.5, fold change ≥2) in expression of genes in Caco-2 cells. The number of differentially expressed genes in Caco-2 cells as a function of carbon source is summarized in Table 1. During co-incubation with *B. breve* SC95, the expression of 12 Caco-2 cell genes was upregulated and 61 genes were downregulated by HMO compared to GLU, while 196 genes were upregulated and 144 genes were downregulated by HMO compared to LAC. Comparison between GLU vs LAC grown samples of *B. breve* SC95 yielded 96 up-regulated genes and 426 downregulated genes by LAC.

**Table 1:** Summary of the RNA-Seq gene expression analysis. Numbers of genes that had significant expression changes in Caco-2 cells

Strain	Comparison	Expression change^a^
*B. breve* SC95	GLU vs HMO	12 genes upregulated and 61 genes downregulated by HMO
	LAC vs HMO	196 genes upregulated and 144 genes downregulated by HMO
	GLU vs LAC	96 genes upregulated and 426 genes downregulated by LAC
*B. infantis* ATCC 15697	GLU vs HMO	107 genes upregulated and 178 genes downregulated by HMO
	LAC vs HMO	21 genes upregulated and 37 genes downregulated by HMO
	GLU vs LAC	97 genes upregulated and 48 genes downregulated by LAC

^a^Genes with statistically significant changes in expression

Co-incubation of Caco-2 cells with *B. infantis* ATCC 15697 also modified Caco-2 gene expression as a function of carbon source (Table 1). There were 107 genes upregulated and 178 genes downregulated by HMO compared to GLU and upregulation of 21 genes and downregulation of 37 genes by HMO compared to LAC. When comparing GLU vs LAC grown *B. infantis* ATCC 15697, 148 genes were upregulated and 97 genes were downregulated by LAC (Table 1). List of these genes with significant changes in expression and their RPKM values are provided in Additional file 1 and Additional file 2.

**Fig. 2:**
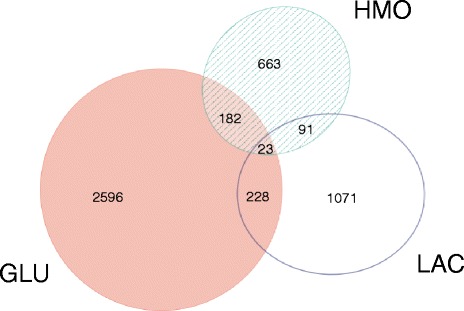
Number of Caco-2 genes differentially expressed between *B. infantis* ATCC 15697 and *B. breve* SC95 in response to different substrates (GLU = glucose, LAC = lactose, HMO = human milk oligosaccharides). Venn diagram prepared using EulerAPE [62]

To compare the differential effects of bifidobacterial strain and carbon source on the Caco-2 cell transcriptome, between-strain differential gene expression was examined for each of the three carbon sources. The number of Caco-2 genes differentially expressed in response to co-incubation with *B. infantis* compared with *B. breve* for each carbon source is listed in Fig. 2. For example, there are 2596 Caco-2 genes that are differentially expressed in response to GLU-fed *B. infantis* compared to GLU-fed *B. breve* that are not also differentially expressed in response to HMO- or LAC-fed strains. The carbon source of glucose elicits the biggest bifidobacteria strain effect on Caco-2 gene expression. Meanwhile, the use of HMO as a carbon source appears to reduce the difference between strains. If the choice of carbon source were unimportant, the intersection of the gene expression results (Fig. 2) should be quite high. However, that is not the case. The intersection of the three carbon sources is rather small (Fig. 2), suggesting that the choice of carbon source definitively affects Caco-2 response to different bifidobacteria strains. Thus, the differential response of Caco-2 cells to the two different bifidobacteria strains is highly dependent on the carbon source.

### Predicted consequences of bifidobacterial carbon source on Caco-2 function

To investigate the functional consequences of bifidobacteria carbon source on Caco-2 cell gene expression, functional annotation clustering was performed using DAVID [37] for each input gene list (Table 1). These results were filtered to obtain terms with statistically significant enrichment for each comparison (Tables 2 and 3). In the co-incubation of *B. breve* SC95 with Caco-2 cells (Table 2), genes that were downregulated by HMO, relative to GLU, were enriched with the annotation term GO:0008009 ~ chemokine activity. Several other annotation terms related to inflammation and immunity were also significantly enriched among genes downregulated by HMO grown *B. breve* SC95 (Table 2). There was no significant enrichment of annotation terms for genes up-regulated by HMO grown, relative to GLU grown, *B. breve* SC95. Likewise, genes downregulated by HMO-fed *B. breve* SC95, relative to LAC-fed, were also enriched with the annotation term “chemokine activity.”

**Table 2:** Enriched annotation terms with significant changes in Caco-2 cells exposed to *B. breve* SC95 grown on different carbon sources

GLU vs HMO comparison: cluster annotation of genes downregulated by HMO		
Enrichment score	Term^1^	Gene count
8.8	GO:0008009 ~ chemokine activity	9
4.2	GO:0005576 ~ extracellular region	19
3.8	GO:0050900 ~ leukocyte migration	5
2.9	GO:0048514 ~ blood vessel morphogenesis	6
2.8	GO:0006915 ~ apoptosis	9
2.7	GO:0051101 ~ regulation of DNA binding	5
2.7	GO:0031328 ~ positive regulation cellular biosynthetic process	10
2.3	GO:0043066 ~ negative regulation of apoptosis	7
2.1	GO:0031349 ~ positive regulation of defense response	4
1.9	GO:0002763 ~ positive regulation myeloid leukocyte differentiation	3
HMO vs LAC comparison: cluster annotation of genes upregulated by HMO		
Enrichment score	Term^1^	Gene count
9.5	GO:0045449 ~ regulation of transcription	58
4.6	IPR015880:Zinc finger, C2H2-like	24
2.8	IPR012287:Homeodomain-related	10
HMO vs LAC comparison: cluster annotation of genes downregulated by HMO		
Enrichment score	Term^1^	Gene count
6.4	GO:0008009 ~ chemokine activity	8
5.9	GO:0042981 ~ regulation of apoptosis	21
5.0	GO:0043066 ~ negative regulation of apoptosis	13
4.2	GO:0031328 ~ positive regulation cellular biosynthetic process	18
3.3	IPR004827:Basic-leucine zipper (bZIP) transcription factor	6
3.1	GO:0051272 ~ positive regulation of cell motion	7
3.1	GO:0051674 ~ localization of cell	10
2.7	GO:0005840 ~ ribosome	8
2.7	GO:0051090 ~ regulation of transcription factor activity	6
2.6	GO:0001525 ~ angiogenesis	7
2.4	GO:0010557 ~ positive regulation macromolecule biosynthesis	14

Term^1^: Annotation terms with enrichment score ≥1.3, p value (EASE score) ≤0.05 and globally corrected enrichment Benjamini *p*-value (to control family-wide false discovery rate) ≤0.05 were selected

**Table 3:** Enriched annotation terms with significant changes in Caco-2 cells exposed to *B. infantis* ATCC 15697 grown on different carbon sources

GLU vs HMO cluster annotation of genes downregulated by HMO		
Enrichment score	Term	Gene count
3.51	IPR000558:Histone H2B	4
3.16	GO:0032993 ~ protein-DNA complex	8
3.07	GO:0003677 ~ DNA binding	33
HMO vs LAC cluster annotation of genes downregulated by HMO		
Enrichment score	Term	Gene count
5.68	GO:0008009 ~ chemokine activity	6
2.63	GO:0005615 ~ extracellular space	8

Functional enrichment analysis of the Caco-2 cell genes modulated by *B. infantis* (Table 3) also showed the modulation of “chemokine activity” by HMO. Relative to LAC-fed *B. infantis*, HMO-fed *B. infantis* downregulated Caco-2 cell genes associated with “chemokine activity.” However, this difference was not seen in the comparison between HMO-fed and GLU-fed *B. infantis*. There was no significant enrichment of annotation terms for genes upregulated by HMO-fed *B. infantis* compared to either GLU- or LAC-fed. In summary, consumption of HMO by two different bifidobacterial strains was associated with downregulation of inflammation-related signaling in Caco-2 cells.

### Expression of inflammation related genes is reduced by HMO-grown bifidobacteria

**Fig. 3:**
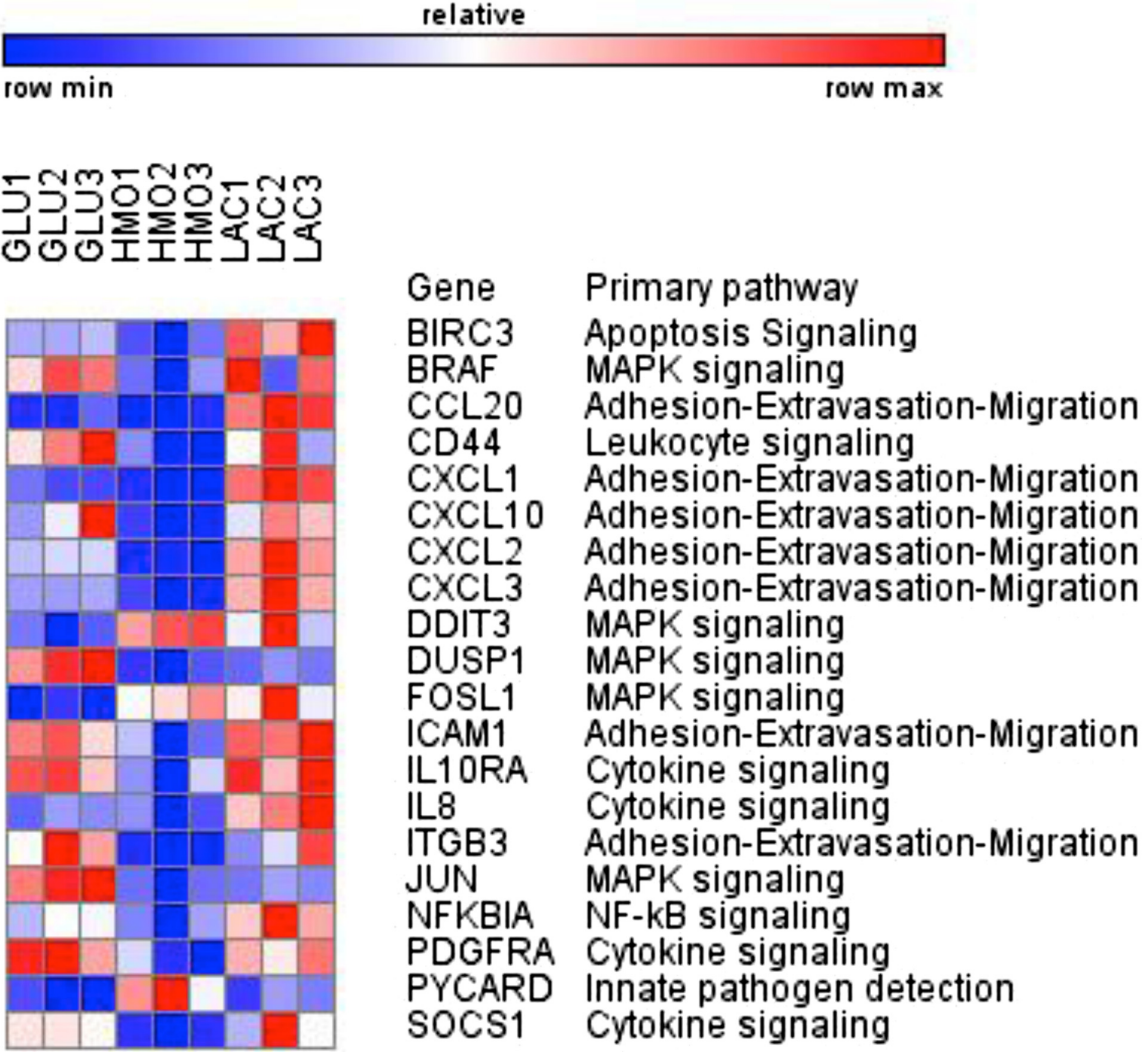
Heat map of inflammation related genes with significant changes in expression in Caco2 cells exposed *B. infantis* ATCC 15697 grown in GLU, HMO or LAC. Three replicates are shown for each sugar

**Fig. 4:**
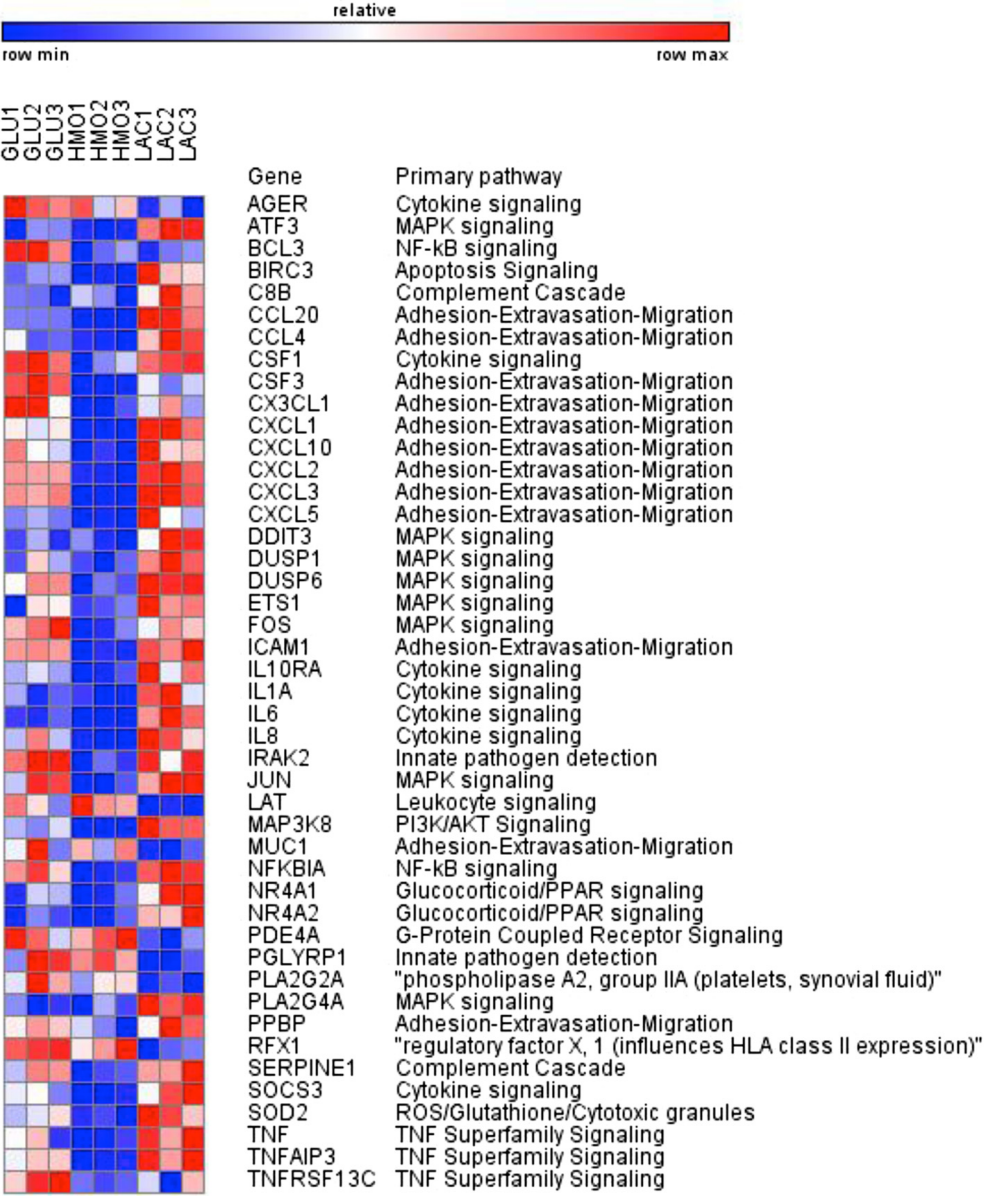
Heat map of inflammation related genes with significant changes in expression in Caco2 cells exposed *B.breve* SC95 grown in GLU, HMO or LAC. Three replicates are shown for each sugar

Heat maps summarizing significantly differentially expressed inflammation–related genes in Caco-2 cells co-incubated with *B. infantis* and *B. breve* are shown in Figs. 3 and 4, respectively. Nearly all of these genes are downregulated by HMO feeding relative to either GLU or LAC or both.

**Fig. 5:**
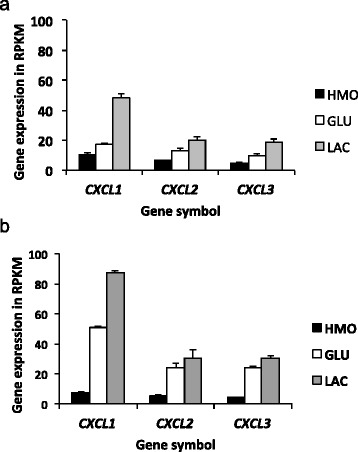
Expression of CXCL1, CXCL2 and CXCL3 genes in **a** *B. infantis* ATCC 15697 grown on HMO, GLU or LAC, and **b** *B. breve* SC95 grown on HMO, GLU or LAC. Expression of these genes were significantly downregulated by HMO grown bifidobacteria

Necrotizing enterocolitis (NEC) is a very common emergency occurring in pre-term infants. Previous studies have shown that NEC can be prevented by probiotics perhaps in part because they modulate the immune mediated gene expression in enterocytes [38]. Given that the functional enrichment analyses and the heat maps (Figs. 3 and 4) pointed to chemokine-related changes in gene expression in the Caco-2 cells exposed to bifidobacteria grown in different carbon sources, we specifically investigated genes involved in inflammation. Expression of *CXCL1, CXCL2* and *CXCL3* were downregulated by HMO feeding of both strains of bifidobacteria (Fig. 5a and b). Another cell adhesion and chemotaxis target gene that has shown higher expression in NEC is *ICAM1* [39] and interestingly this gene was significantly downregulated in Caco-2 cells exposed to both *B. infantis* ATCC 15697 and *B. breve SC95* grown in HMO (Figs. 3 and 4).

Aberrant modulation of gut bacteria, such as bifidobacteria, has been implicated in inflammatory bowel disease (IBD) [40, 41]. Therefore, we screened candidate genes for ulcerative colitis (UC) and Crohn’s disease for significant changes in expression in the RNA-Seq data. Numerous candidate genes were downregulated in Caco-2 cells exposed to *B. infantis* ATCC 15697 and/or *B. breve SC95* grown on HMO (Table 4). *ICAM1,* which is known to be highly expressed in both Crohn’s disease and UC relative to controls [42], was downregulated in Caco-2 cells exposed to either HMO-fed bifidobacterial strain.

**Table 4:** Known candidate genes for Crohn’s disease and IBD with significant changes of expression in Caco-2 cells exposed to bifidobacteria strains grown on different carbon sources

Comparison	*B. infantis*	*B. breve*
GLU vs HMO		
Upregulated by HMO	LTB	None
	TNNI2	
Downregulated by HMO	LRRK2	FOS
	SOCS1	ICAM1
		TNFAIP3
		TNFRSF18
LAC vs HMO		
Upregulated by HMO	None	DOK3
		LAT
		MUC1
		RASIP1
Downregulated by HMO	ICAM1	FOS
	LRRK2	ICAM1
		LTB
		MAP3K8
		TNF
		TNFAIP3
		TNFRSF18

Candidate genes for Crohn’s disease: *LTB, LRRK2, ICAM1, LAT, MUC1, RASIP1, TNF*

Candidate genes for Ulcerative colitis: *TNNI2, SOCS1, FOS, TNFAIP3, TNFRSF18, DOK3, MUC1, MAP3K8*

## Discussion

Commensal bifidobacteria have been associated with regulation of intestinal inflammation [38, 43, 44]. Bifidobacteria are normal residents of the human intestine, commonly found in the infant gut and particularly enriched in the intestine of breastfed infants [14]. *B. infantis* and *B. breve* are often dominant members of the breastfed infant gut microbiota [30] and have been linked to a modulatory role in intestinal inflammation and regulation of the immune response at the gut associated lymphoid tissue [45]. It is well accepted that bifidobacteria overrepresentation in nursing infants brings benefits to the neonate [17].

One of the major driving forces that underlie bifidobacterial predominance in infants is the ability to consume HMOs. In a previous study, we demonstrated that growth on HMOs significantly increased the binding of *B. infantis*, but not *B. bifidum*, to intestinal cells [26]. Consistent with that study, we found that HMO-grown *B. infantis* showed significant adherence to Caco-2 cells. More recently, we have characterized unique strains of *B. breve* that grow well on HMO [19] which led us to hypothesize that these strains might also exhibit an HMO-induced binding phenotype. However, unlike *B. infantis*, *B. breve* SC95 showed low percentage of binding with Caco-2 cells, regardless of the growth medium. Previous studies demonstrate that the adherence of *B. breve* to Caco-2 cells is strain-specific and that adherence of a high-adhering strain of *B. breve* is almost totally abolished with trypsin or pronase treatment of the culture [46]. In the current protocol we have discarded the spent culture and this may explain the low binding percentage of *B. breve* SC95*.* An interesting future study design would be to include the spent culture of bifidobacteria in Caco-2 binding assays. The transcriptomic changes of Caco-2 cells evoked by *B. breve* may result from the constitutive expression of surface structures or the production of soluble factors. There is evidence that bifidobacteria exert an indirect effect on intestinal inflammation, due to the release of soluble factors that reduce inflammation [38].

Taken together, both strain and carbon source affect the binding affinity of bifidobacteria to IECs. In a previous study, we found that HMO-grown *B. infantis* increased the expression of genes involved in promoting integrity of the barrier function [26]. However, that study was a targeted approach in which only a few genes were probed. Here, we employed a high-throughput sequencing method to obtain an overview of all gene expression in Caco-2 cells upon incubation with *B. infantis* ATCC 15697 and *B. breve* SC 95 grown in different carbon sources. With each bifidobacterial strain, the supplied carbon source—GLU, LAC, or HMO—significantly affected gene expression in Caco-2 cells. Interestingly, even though the adhesive ability of *B. breve* was low, it still elicited a significant transcriptional response in Caco-2 cells, altering even more genes than *B. infantis.* These observations suggest that mechanisms additional to that of direct binding of *B. breve* are important to promote gene expression changes in Caco-2 cells. Indirect mechanisms, such as the production of soluble factors by *B. breve* during interaction with IECs, may underlie the gene expression changes and it should be investigated.

In a non-hypothesis driven assessment of Caco-2 gene expression in response to bifidobacteria grown on different carbon sources, we found that growth on HMOs substrate reduced inflammation-related signaling. The reduced expression of genes related to chemokine activity in the presence of HMO-grown bifidobacteria occurred independent of bifidobacterial strain, suggesting that prebiotic HMO primes bifidobacteria to elicit an anti-inflammatory state in IECs. RNA-Seq analysis showed significant downregulation of three chemokines in CXCL family: *CXCL1, CXCL2* and *CXCL3.* These chemokines play an active role in development of gastrointestinal diseases marked by inflammatory response such as gastritis, necrotizing enterocolitis (NEC), ileitis, ulcerative colitis [47] and inflammatory bowel diseases (IBD) [48]. Mouse studies have showed an association between higher incidence of necrotizing enterocolitis and elevated intestinal expression of *CXCL1* mRNA [49]. A recent study where *B. infantis* was administered to mouse pups that are prone to NEC has shown significant downregulation of *CXCL1* gene in the intestinal epithelium [43]. The *in vivo* blocking of *CXCL2* has been shown to alleviate inflammation related bowel injuries [50]. *CXCL1, CXCL2* and *CXCL3* are positively associated with tumor associated angiogenesis and depletion of these three chemokine factors have inhibited the tumor growth in mice [51]. 

Given the effect of HMO-grown bifidobacteria on inflammatory related signaling in Caco-2 cells in this study, we specifically reviewed the Caco-2 transcriptome data in the context of known candidate genes associated with NEC, Crohn’s diseases and ulcerative colitis. Several candidate genes associated with these bowel diseases are significantly regulated by HMO-grown bifidobacteria (Table 4). Expression of intracellular adhesion molecule-1 (*ICAM1*) was significantly downregulated by *B. breve* and *B. infantis* grown in HMO. *ICAM1*, located in the luminal surface of the intestinal epithelium, is a ligand for neutrophils. *ICAM1* facilitates the transepithelial migration of neutrophils and their accumulation in the luminal surface of the intestine, which contributes to mucosal injury leading to conditions such as ulcerative colitis [52]. Therefore the decreased expression of *ICAM1* by *B. breve* and *B. infantis* grown in HMO may aid to reduce the risk of NEC in infants and inflammatory bowel disease in adults. These results highlight the therapeutic potential to prevent or possibly ameliorate IBD with synbiotics that include both bifidobacteria and milk oligosaccharides.

## Conclusions

This study presents an initial assessment of the transcriptomic changes of intestinal epithelial cells evoked by commensal bifidobacteria grown on different sugars. We provide evidence that consumption of HMOs, which are abundant in the gastrointestinal tract of nursing infants, promotes a beneficial interaction between bifidobacteria and the intestinal epithelium. Consumption of free milk glycans by bifidobacteria leads to reduced expression of inflammatory genes, contributing to maintenance of the integrity of the intestinal mucosa. Our study represents a major step towards a better understanding of bifidobacteria-host interactions that take place in the infant gut, which can be applied to the use of bifidobacteria as probiotics to promote gut health. Moreover, the results presented herein provide support for the use of prebiotic HMOs as the best growth substrate for bifidobacteria, not only by supporting their growth to high densities and contributing to niche occupation, but also by inducing protective responses in the host.

## Methods

### Bacterial strains and culture conditions

*Bifidobacterium longum* subsp. *infantis* ATCC 15697 and *B. breve* SC95 were routinely grown for 48 h anaerobically at 37 °C in the semisynthetic de Man-Rogosa-Sharpe (MRS) broth or MRS agar (Becton Dickinson) supplemented with 1 % (wt/vol) L-cysteine. Single colony isolates were inoculated into modified MRS (mMRS) without sugar supplemented with 2 % of filter-sterilized (wt/vol) lactose (LAC) (Sigma Aldrich), 2 % glucose (GLU) (Fisher) or 2 % purified human milk oligosaccharide mixture (HMO) as the sole carbon source. HMO was kindly provided by the laboratory of Dr. Bruce German (Department of Food Science and Technology, University of California, Davis) and purified according to the method described in Gnoth et al. [53].

### Caco-2 cell culture

Enterocyte-like human colon adenocarcinoma (Caco-2) cells were obtained from the American Type Culture Collection (ATCC® HTB37™). These cells undergo spontaneous differentiation during culture, expressing some transporters and metabolic enzymes normally present in the gut [54]. Caco-2 cells were routinely cultured in 75-cm^2^ flasks at 37 °C in a 5 % CO_2_ constant-humidity environment with medium replaced every 2–3 days. Caco-2 cells were grown using in Dulbecco’s modified Eagle medium (DMEM) containing 20 % heat-inactivated fetal calf serum (FBS), 1 % nonessential amino acids, 50 IU/mL penicillin, and 50 μg/mL streptomycin. Cells were subcultured at 80 % confluence by adding 0.25 % trypsin/0.9 mM EDTA solution (Invitrogen, CA). For adhesion and gene expression assays, Caco-2 cells were seeded in 24-well plate (2 cm^2^/well; BD Falcon, Franklin Lakes, NJ) at 1 × 10^5^cells per well. The viable cell number was obtained using a trypan blue and TC20^™^automated cell counter. Caco-2 cell monolayers were used fifteen days after confluence, a time when morphological and functional differentiation is complete [55].

### Adhesion assay

*B. infantis* ATCC 15697 and *B. breve* SC95 from the exponentially grown 48 h-old cultures supplemented with HMO, GLU or LAC were collected by centrifugation (4000 g for 10 min), washed with sterile phosphate-buffered saline (PBS; pH 7.3), and resuspended in DMEM at approximately 1 × 10^8^ cells/mL. For reference purposes (100 % values), 1 ml aliquots of the original bacterial cell suspensions used in the adhesion assay were centrifuged, the cells resuspended in 200 μl trypsin/EDTA plus 200 μl PBS and then frozen and stored at -20 °C until quantification of the bacteria. *B. infantis* ATCC 15697 and *B. breve* SC95 bacterial suspensions were incubated with a monolayer of fully differentiated Caco-2 cells at 37 °C, 5 % CO_2_ for 2 h. All incubations were performed in biologically independent triplicates. After 2 h of incubation, cell monolayers were gently washed three times with PBS, to remove unbound bacteria, and then detached from the plastic surface by incubation with 200 μl trypsin/EDTA per well (10 min, 37 °C). To perform quantification of adherent bacteria, cell suspensions were incubated at 37 °C for 30 min in Gram-positive lysis buffer (20 mM Tris–HCl, 2 mM sodium EDTA, 1.2 % Triton X-100 and 20 mg/ml lysozyme). Quantification of adherent bacteria was performed by quantitative PCR targeting the 16S rRNA gene. The primers employed were as follows: Bif F (5′-TCGCGTCTGGTGTGAAAG-3′) and Bif R (5′-CCACATCCAGCGTCCAC-3′) for *B. infantis* [26]. *B. breve* was analyzed using primers BiBRE-F (5′- CCGGATGCTCCATCACAC-3′) and BiBRER (5′- ACAAAGTGCCTTGCTCCCT-3′). A standard curve for quantification of bifidobacterial strains was generated from serial dilutions of bacterial DNA and used to calculate numbers of bacterial copies. Estimates of the number of bifidobacterial genome copies in the standard were based on a genome size (1.75–2.8 Mb) of the individual strain. Quantitative PCR was performed in a 7500 Fast Real-Time PCR System (Applied Biosystems, CA) using SYBR Green fluorophore. The PCR reactions and melting curves were performed in 20 μl containing 1 μl of each primer, 10 μl SYBR Green PCR Master Mix 2× (Applied Biosystems, CA), and 2 μl of bacterial DNA. The PCR reaction was incubated at 95 °C for 10 min, followed by 40 cycles consisting of 20 s at 95 °C, 20 s 56 °C, and 30 s at 60 °C. Bacterial adhesion was expressed as the number of adherent bacteria divided by total number of bacteria added, multiplied by 100 [26].

### RNA-Seq experiment and data analysis

*B. infantis* ATCC 15697 and *B. breve* SC95 cells were incubated with fully differentiated Caco-2 cell monolayers as described above for the adhesion assay. All incubations were performed in biologically independent triplicates that consisted of independent bacterial cultures and Caco-2 cells that are of different passages. After 2 h of incubation, Caco-2 cell monolayers were gently washed three times with PBS and RNA was extracted from Caco-2 cells using the Trizol method according to the manufacturer’s instructions (Invitrogen, Carlsbad, CA). RNA was quantified by an ND-1000 spectrophotometer (Fisher Thermo, Wilmington, MA), and the quality and integrity was assessed by the spectrophotometer 260/280 ratio, gel electrophoresis and capillary electrophoresis with an Experion bio-analyzer (Bio-Rad, Hercules, CA).

Gene expression analysis was conducted using Illumina RNA-Seq technology. Messenger RNA was isolated and purified using a TruSeq RNA Sample Prep Kit v2 (Illumina, San Diego, CA). The fragments were sequenced at Vincent J. Coates Genomics Sequencing Laboratory of University of California Berkeley using the Illumina HiSeq2000. Sequence reads of 100 bp were assembled and analyzed in RNA-Seq and expression analysis application of CLC Genomics Workbench 5.5.1 (CLC Bio, Aarhus, Denmark). The human genome, *H. sapiens* Build 37.1 (ftp://ftp.ncbi.nih.gov/genomes/H_sapiens/ARCHIVE/BUILD.37.1/) was utilized as the reference genome for the assembly. The following criteria were used to filter the unique sequence reads: minimum length fraction of 0.9; minimum similarity fraction of 0.8; maximum number of two mismatches. Data were normalized by calculating the reads per kilo base per million mapped reads (RPKM = total exon reads/mapped reads in millions × exon length in kb) [56] for each gene and annotated with Ensembl human genome assembly GRCh37.p11 (57,412 total genes).

Significant gene expression changes in Caco-2 cells exposed to *B. infantis* ATCC 15697 or *B. breve* SC95 grown in HMO, GLU or LAC were analyzed using t-tests on log_2_ transformed data (0.5 was added to each number before log transformation to deal with zero counts). Analyses were conducted, 1) on the same strain grown on different sugars 2) between the two strains grown on same sugars. Genes with *p*-value ≤0.05, FDR q ≤ 0.5 and fold change ≥2 were considered to be statistically significant.

Genes with significant change in expression levels were further analyzed using the functional analysis clustering tool of The Database for Annotation, Visualization and Integrated Discovery (DAVID) v6.7 [57]. High classification stringency, enrichment score ≥1.3, *p*-value (EASE score) ≤0.05 and globally corrected enrichment Benjamini *p*-value (to control for family-wide false discovery rate) ≤0.05 were the statistical parameters used to cluster functionally similar annotation terms associated with the input gene list.

Inflammation is a valuable protection system in the body against harmful external and internal stimuli [58]. However the dysfunctional regulation of the intestinal immune system is a contributory factor to many diseases such as necrotizing enterocolitis (NEC) [39], inflammatory bowel disease (IBD) [59], celiac disease, and Crohn’s disease [60]. Loza et al. assembled 1027 inflammation related genes using literature survey and Ingenuity pathway analysis [58]. This gene list was used to extract the inflammation related genes that showed significant changes in expression in our RNA-Seq analysis. Genes with significant changes in expression in the RNA-Seq experiment were also compared to lists of genes associated with Crohn’s disease [61], inflammatory bowel disease and ulcerative colitis [41].

### Availability of supporting data

The raw sequencing data and processed data have been deposited in NCBI’s GEO database. The RNA-Seq experiments involving the co-incubation of Caco-2 cells with *B. infantis* and *B. breve* are deposited as accessions GSE63950 and GSE64017, respectively.

## Additional files

Additional file 1:
**A Microsoft Excel file that contains spreadsheets of Caco-2 genes differentially expressed in response to** ***B. infantis*** **grown on GLU, LAC, or HMO.** (XLSX 86 kb) Additional file 2:
**A Microsoft Excel file that contains spreadsheets of Caco-2 genes differentially expressed in response to** ***B. breve*** **grown on GLU, LAC, or HMO.** (XLSX 177 kb)

## Abbreviations

HMO: Human milk oligosaccharides
GLU: Glucose
LAC: Lactose
RNA-Seq: RNA sequencing
IECs: Intestinal epithelial cells
